# Development of a pediatric physiologically-based pharmacokinetic model to support recommended dosing of atezolizumab in children with solid tumors

**DOI:** 10.3389/fphar.2022.974423

**Published:** 2022-09-26

**Authors:** Weize Huang, Felix Stader, Phyllis Chan, Colby S. Shemesh, Yuan Chen, Katherine L. Gill, Hannah M. Jones, Linzhong Li, Gianluca Rossato, Benjamin Wu, Jin Y. Jin, Pascal Chanu

**Affiliations:** ^1^ Genentech Inc, South San Francisco, CA, United States; ^2^ Certara UK Limited, Sheffield, United Kingdom; ^3^ F. Hoffmann-La Roche Ltd, Basel, Switzerland

**Keywords:** alveolar soft part sarcoma, atezolizumab, physiologically-based pharmacokinetic (PBPK) modeling, pediatric extrapolation, pediatric oncology, solid tumor, clinical pharmacology, quantitative pharmacology

## Abstract

**Background:** Atezolizumab has been studied in multiple indications for both pediatric and adult patient populations. Generally, clinical studies enrolling pediatric patients may not collect sufficient pharmacokinetic data to characterize the drug exposure and disposition because of operational, ethical, and logistical challenges including burden to children and blood sample volume limitations. Therefore, mechanistic modeling and simulation may serve as a tool to predict and understand the drug exposure in pediatric patients.

**Objective:** To use mechanistic physiologically-based pharmacokinetic (PBPK) modeling to predict atezolizumab exposure at a dose of 15 mg/kg (max 1,200 mg) in pediatric patients to support dose rationalization and label recommendations.

**Methods:** A minimal mechanistic PBPK model was used which incorporated age-dependent changes in physiology and biochemistry that are related to atezolizumab disposition such as endogenous IgG concentration and lymph flow. The PBPK model was developed using both *in vitro* data and clinically observed data in adults and was verified across dose levels obtained from a phase I and multiple phase III studies in both pediatric patients and adults. The verified model was then used to generate PK predictions for pediatric and adult subjects ranging from 2- to 29-year-old.

**Results:** Individualized verification in children and in adults showed that the simulated concentrations of atezolizumab were comparable (76% within two-fold and 90% within three-fold, respectively) to the observed data with no bias for either over- or under-prediction. Applying the verified model, the predicted exposure metrics including C_min_, C_max_, and AUC_tau_ were consistent between pediatric and adult patients with a geometric mean of pediatric exposure metrics between 0.8- to 1.25-fold of the values in adults.

**Conclusion:** The results show that a 15 mg/kg (max 1,200 mg) atezolizumab dose administered intravenously in pediatric patients provides comparable atezolizumab exposure to a dose of 1,200 mg in adults. This suggests that a dose of 15 mg/kg will provide adequate and effective atezolizumab exposure in pediatric patients from 2- to 18-year-old.

## Introduction

Atezolizumab is a humanized immunoglobulin (IgG) 1 monoclonal antibody (mAb) that targets human programmed death−ligand 1 (PD-L1) expressed on immune cells and tumor cells, and blocks program death protein 1 (PD-1) mediated inhibitory signals (Akinleye and Rasool, 2019; FDA, 2021). Atezolizumab has been approved for use in multiple indications (e.g., small cell and non-small cell lung cancer, hepatocellular carcinoma, urothelial carcinoma, melanoma) across several countries due to its demonstrated anti-tumor activity and acceptable safety profile (EMA, 2021; FDA, 2021). In adults, the pharmacokinetic (PK) profile of atezolizumab exhibited linearity (for at least 21 days after a single IV dose) over a dose range of 1 mg/kg to 20 mg/kg, with a systemic clearance (CL) of 0.2 L/day, a volume of distribution at steady state (V_SS_) of 6.91 L, and a terminal half-life (t_1/2_) of 27 days (Herbst et al., 2014; Stroh et al., 2017; FDA, 2021). Demographics and baseline characteristics including body weight, sex, albumin levels, tumor burden, and treatment-emergent anti-drug antibody (ADA) status are statistically significant covariates for the PK parameters of atezolizumab. Nonetheless, these factors show no clinically significant effect on systemic exposure of atezolizumab and hence require no dose adjustment (FDA, 2021). In addition, multiple studies have shown that atezolizumab has flat exposure-efficacy and exposure-safety relationships across multiple indications and patient populations (Stroh et al., 2017; Morrissey et al., 2019; Liu et al., 2022). Atezolizumab has also been studied in pediatric patients (Shemesh et al., 2019; Geoerger et al., 2020). Following 15 mg/kg (maximum dose of 1,200 mg) once every 3 weeks (Q3W), atezolizumab steady-state exposure (AUC) in 12- to 18-year-old pediatric patients was comparable to that of adult patients who received 1,200 mg Q3W, while atezolizumab exposure trended lower in pediatric patients less than 12-year-old, although data are relatively limited (Shemesh et al., 2019; FDA, 2021).

Recently, atezolizumab was tested in patients with alveolar soft part sarcoma (ASPS). Preclinical models show that the PD-1/PD-L1 axis likely contributes to immunosuppression in ASPS, and isolated reports in ASPS patients treated with anti PD-1/PD-L1 agents showed complete responders with a subset of patients remaining disease-free for several years (Groisberg et al., 2017; Conley et al., 2018; Raj et al., 2018; Wilky et al., 2019). This study is a small multicenter open-label single-arm phase II trial for ASPS patients aged ≥ 2 years old. It includes patients with newly-diagnosed, unresectable, and metastatic disease (Naqash et al., 2021). In this study, atezolizumab was administered intravenously (IV) at a fixed dose of 1,200 mg in adults or at 15 mg/kg (1,200 mg maximum) Q3W in pediatric patients aged ≥ 2 years old. However, PK data were not obtained due to operational, ethical, and logistical challenges.

To better understand the PK of atezolizumab in pediatric patients, mechanistic PBPK modeling was used as it provides the advantage of incorporating age-dependent changes in physiology and biochemistry that are related to drug disposition (Edginton et al., 2006; Johnson et al., 2006). This approach has been verified with many small molecules (Huang et al., 2017; Lutz et al., 2021; Sinha et al., 2021; Zhu et al., 2021) and several therapeutic proteins (Li et al., 2014; Pan et al., 2020). In this study, we first developed an adult PBPK model using a combination of *in vitro* data and clinical human PK data after a single dose of 0.3 mg/kg and 20 mg/kg of atezolizumab (Herbst et al., 2014) and verified the model against other dose levels (Herbst et al., 2014) and data from additional studies (Stroh et al., 2017). Then, we implemented age-dependent maturation (Pan et al., 2020) and verified the pediatric model using individual PK data from 2- to 18-year-old patients (Shemesh et al., 2019; Geoerger et al., 2020). Finally, we used the verified model to generate simulations that enabled comparison of the atezolizumab PK across different age groups following 15 mg/kg atezolizumab to investigate exposure changes as pediatric subjects grow. The mechanistic PBPK model-based simulations allowed us to better understand atezolizumab exposure in children to support dose rationale and potentially label recommendations without sampling burden to pediatric patients for additional PK data.

## Material and methods

### Model structure and atezolizumab data sources

**FIGURE 1:**
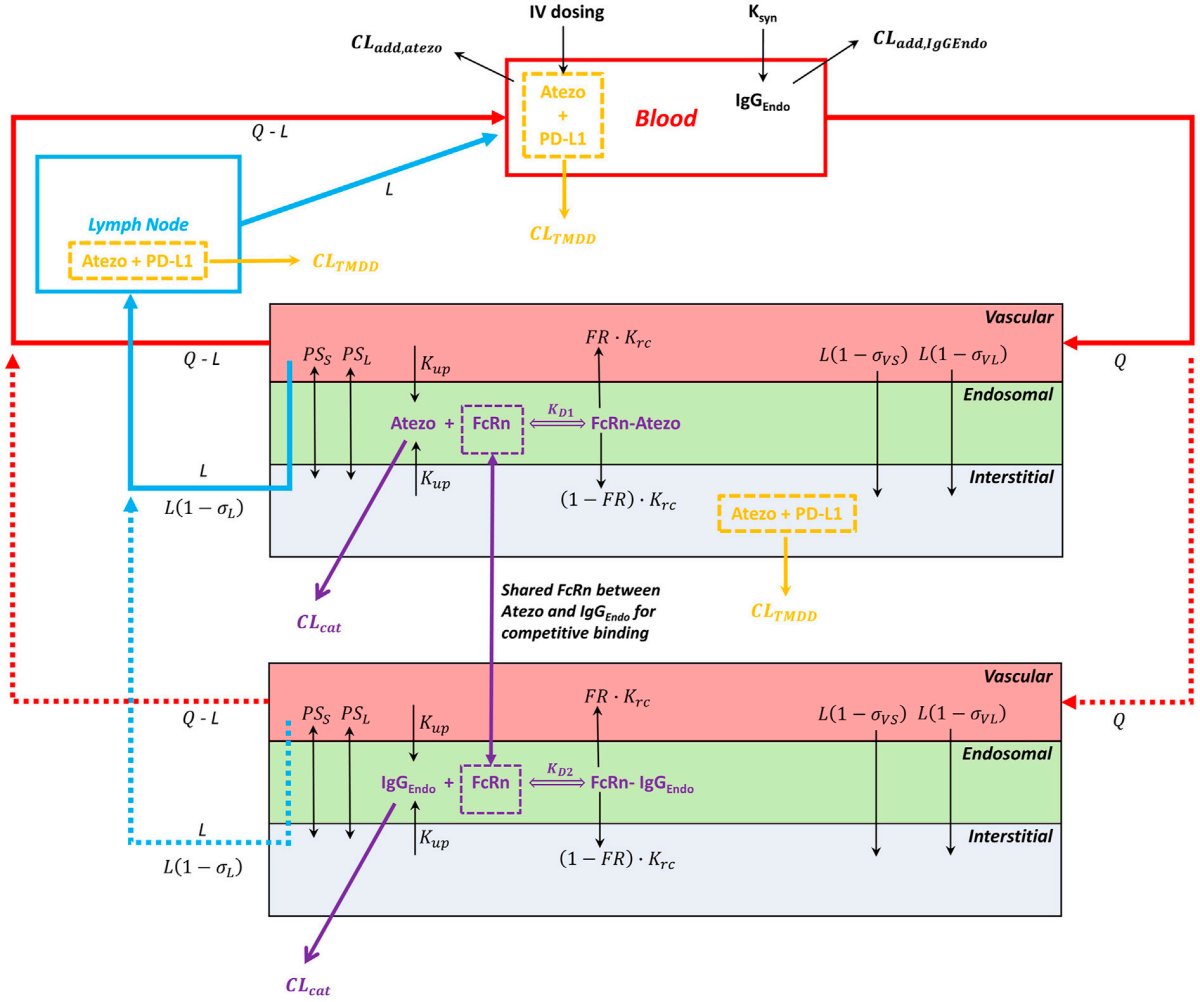
Minimal PBPK model structure and key mechanisms for atezolizumab disposition. The model consists of the blood, lymph node, and lumped tissue compartments. The lumped tissue includes vascular, endosomal, interstitial, and intracellular (not shown) compartments. The 1:1 atezolizumab-FcRn binding at pH 6.0 in the endosomal compartment was considered. The additional clearance (CL_add_) is included in the blood compartment. The TMDD is included in the blood compartment, the lymph node compartment, and the interstitial compartment of the lumped tissue. Abbreviations for model parameters are as follows: CL_add_,_atezo_, additional clearance for atezolizumab; CL_add,IgG,endo_, additional clearance for endogenous IgG; CL_TMDD_, clearance for atezolizumab *via* binding to the target PD-L1; CL_cat_, the intrinsic catabolic clearance of atezolizumab or endogenous IgG that are not bound to FcRn; FcRn, neonatal Fc receptor; K_D1_, equilibrium dissociation constant for atezolizumab-FcRn complex; K_D2_, equilibrium dissociation constant for endogenous IgG-FcRn complex; K_rc_, endosomal recycling rate; K_up_, endosomal uptake rate *via* fluid phase endocytosis; FR, fraction recycled of FcRn-bound atezolizumab or endogenous IgG; PS_s_, permeability surface area product for small pores; PS_l_, permeability surface area product for large pores; σ_vs_, vascular reflection coefficient through small pores; σ_vl_, vascular reflection coefficient through large pores; σ_l_, lymphatic reflection coefficient; L, lymph flow rate; Q, blood flow rate.

**FIGURE 2:**
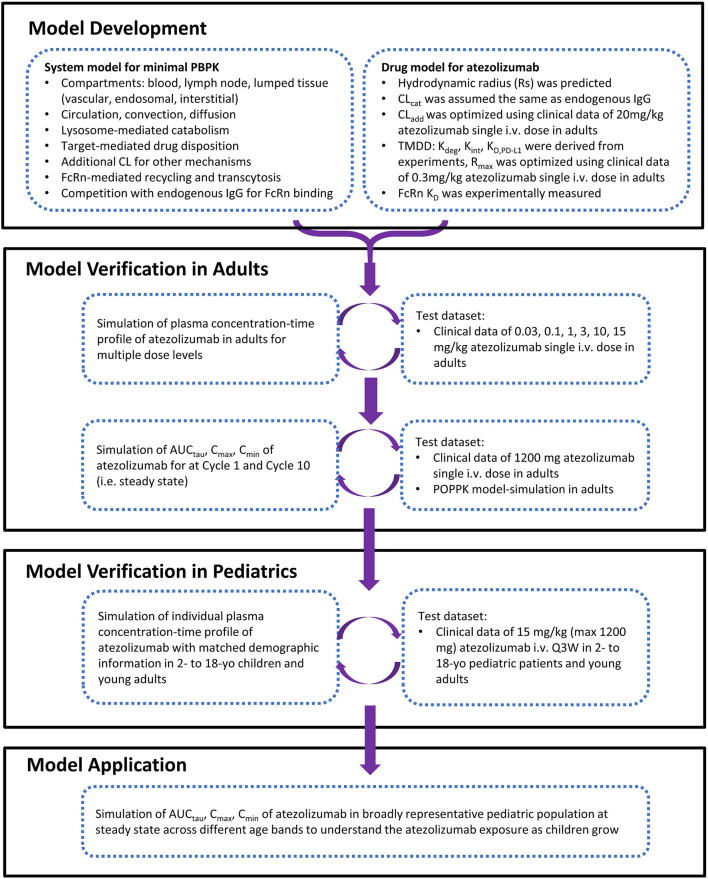
The strategy and workflow of model development, verification, and application for atezolizumab in pediatrics and adult.

A minimal mechanistic PBPK model structure within the Simcyp Simulator version 20R1 (Simcyp Limited, Certara, Sheffield, United Kingdom) was used for atezolizumab. This model structure has been verified previously to capture the systemic disposition of multiple antibody therapeutics (Li et al., 2014; Pan et al., 2020). It also allowed redefinition of the physiology of virtual subjects/population over the length of the simulation for pediatric populations. Briefly, the minimal PBPK model structure contains six compartments: 1) blood; 2) lymph node; and, a lumped tissue that is subdivided into 3) the vascular space, 4) the endosomal space, 5) the interstitial space, and 6) the intracellular space. The model structure with mechanistic pathways is depicted in Figure 1 [adapted from (Li et al., 2014)] and related equations were previously described (Mager and Krzyzanski, 2005; Li et al., 2014; Gill et al., 2016; Pan et al., 2020). The clinical data of atezolizumab used in this model are mainly from three studies (Herbst et al., 2014; Stroh et al., 2017; Shemesh et al., 2019). The overall modeling strategy is illustrated in Figure 2 [adapted from (Huang et al., 2020)].

### Atezolizumab physiologically-based pharmacokinetic model development in adults

In this model, atezolizumab was administered intravenously. The distribution of atezolizumab was governed by circulation *via* blood and lymph flow, diffusion, and convection through pores in the endothelial wall, as well as transcytosis driven by endosomal uptake with subsequent binding to the neonatal Fc receptor (FcRn) and recycling to the vascular or interstitial compartments. The convection and diffusion of atezolizumab was predicted using the 2-pore hypothesis (Rippe and Haraldsson, 1987; Gill et al., 2016), which took into account the hydrodynamic radius (R_s_) of atezolizumab and size of the pores of the lumped tissue (i.e., the weighted average of pore sizes in all specific organs/tissues in the Simcyp full-body PBPK model). The parameter values of vascular reflection coefficient through small and large pores (σ_v,s_ and σ_v,l_) and permeability surface area product for small and large pores (PS_s_, and PS_l_) in the lumped tissue were calculated using a previously-described method (Rippe and Haraldsson, 1987; Aukland and Reed, 1993). Similar to many full-length IgG-like mAbs, atezolizumab also undergoes non-specific pinocytosis [i.e., endosomal uptake (K_up_), that eventually forms the acidified endosomal compartment (pH 6.0) where atezolizumab binds to FcRn [K_D_ value of 650 nM (Chung et al., 2019) with 1:1 binding stoichiometry] for protection from catabolic degradation (CL_cat_) and recycling (K_rc_). During this process, endogenous IgG and atezolizumab were modeled simultaneously to enable competition for FcRn binding in the endosomal space (Li et al., 2014). Endogenous IgG had a predicted Rs of 5.0 nm, a measured FcRn K_D_ value of 728 nM (Suzuki et al., 2010), a synthesis rate (K_syn_) of 0.64 μM/h, an experimentally-based K_up_ of 0.0298 L/h (Haigler et al., 1979), an optimized K_rc_ value of 3.13 L/h, a lysosome-mediated CL_cat_ value of 0.0175 L/h (Li et al., 2014), and a CL_add_ value of 0.0017 L/h which encompassed elimination pathways such as Fcγ receptor-mediated phagocytosis and degradation in non-FcRn-expressing cells. This model was calibrated to capture the known kinetics of endogenous IgG, the observed mean plasma endogenous IgG concentration around 80 µM in adults, and the reduction of endogenous IgG level after IVIG administration and in FcRn deficient subjects. These FcRn-mediated mechanisms are particularly relevant to the pediatric population as endogenous IgG level is dependent on age (Allansmith et al., 1968; Aksu et al., 2006; Pan et al., 2020), in contrast to the relative constant level around 80 µM in the adult population (Waldmann and Strober, 1969; Pan et al., 2020). For atezolizumab, the K_up_, K_rc_, and fraction recycled (FR) values were assumed to be the same as for endogenous IgG, resulting in an estimated V_SS_ value of 0.066 L/kg.

Three atezolizumab clearance pathways were implemented: 1) target-mediated drug disposition (TMDD) through PD-L1 binding and subsequent elimination; 2) catabolism in the lysosome (CL_cat_); and 3) unspecified additional systemic clearance (CL_add_). The clearance pathways were optimized using a mixed bottom-up and top-down approach (i.e., middle-out) leveraging both *in vitro* data and clinical data after high dose (minimal TMDD) and low dose (significant TMDD) treatment (Herbst et al., 2014). In this regard, the initial model used an *in vitro* FcRn K_D_ value for atezolizumab and default CL_cat_ (i.e., same as endogenous IgG) without CL_add_, which resulted in overprediction of systemic exposure (Supplementary Figure S1). Thus, the model was calibrated by adjusting the CL_add_ value to ensure that the simulated plasma atezolizumab concentrations following a single dose of 20 mg/kg (the highest dose level was chosen to ensure the impact of TMDD on the overall clearance was minimal) could successfully recapitulate the observed data in adult patients with solid tumors and hematologic malignancies (Herbst et al., 2014).

As clinical data at dose level <1 mg/kg showed pronounced nonlinear PK (Herbst et al., 2014), the TMDD pathway was also incorporated into the adult atezolizumab PBPK model. Since PD-L1 exists as both a soluble and a membrane-bound receptor on immune cells located in blood, interstitial fluid, and lymph, the TMDD component was included into each of these compartments in the PBPK model. For the target, a median PD-L1 degradation rate constant (K_deg_) of 0.0426 L/h was calculated from values presented in multiple publications (Supplementary Table S1). A PD-L1 expression level (R_max_) value of 0.609 nM was fitted from clinical data in adult patients with solid tumor and hematological malignancy receiving 0.3 mg/kg atezolizumab (Herbst et al., 2014). A PD-L1 K_syn_ value of 2.59 × 10^−5^ μM/h was then calculated from R_max_ × K_deg_. For the binding, a quasi-equilibrium model was used to describe the TMDD for atezolizumab, using a measured internalization rate constant (K_int_) value of 0.606 L/h determined from the percent of PD-L1 remaining at the cell surface as evaluated in the pancreatic cancer cell line BxPC-3 using flow cytometry analysis (Burr et al., 2017). Total PD-L1 was considered dynamic in the model due to its differing internalization and degradation rate constants. Equilibrium binding studies to determine atezolizumab K_D_ for PD-L1 were performed using three lots of atezolizumab and its chimeric derivative, PRO304397, with human PD-L1 expressed on 293 cells (EMA, 2021). Thus, a weighted mean K_D_ value of 0.299 nM was calculated from the reported *in vitro* values (Supplementary Table S2). Overall, four atezolizumab-specific parameters (FcRn K_D_, PD-L1 K_deg_, PD-L1 K_int_, and PD-L1 K_D_) were derived from experiments and two parameters were optimized (CL_add_ and R_max_) using human data. All final model parameters are shown in Table 1.

**TABLE 1:** Parameter values for atezolizumab PBPK model.

Parameter	Value	References
Physicochemical parameters		
Molecular Weight (kDa)	145	FDA, (2021)
Hydrodynamic radius, R_s_ (nm)	5.01	Predicted
B:P	0.55	Default
fu_p_	1	Default
Distribution parameters—minimal PBPK model—1:1 FcRn binding		
K_D1_ (µM)	0.65	Chung et al. (2019)
K_up_ (1/hr)	0.0298	Assumed to be the same as endogenous IgG
K_rc1_ (1/hr)	3.13	Assumed to be the same as endogenous IgG
FR	0.5	Assumed to be the same as endogenous IgG
σ_v,s_	0.9	Calculated
σ_v,l_	0.16	Calculated
PS_s_	0.0017	Calculated
PS_l_	0.0029	Calculated
Elimination parameters		
CL_cat_ (L/hr)	0.0175	Assumed to be the same as endogenous IgG
CL_add_ (L/hr)	0.00693	Optimized
CL_lymph_ (L/hr)	0	No binding to Fcγ receptors and thus no macrophage clearance in the lymph is expected (Inman et al., 2017)
TMDD parameters—target		
PD-L1 R_max_ (µM)	0.000609	Optimized
PD-L1 K_deg_ (1/hr)	0.0426	Median value from meta-analysis (Supplementary Table S1)
PD-L1 K_syn_ (µM/hr)	2.59 × 10^-5^	Calculated
PD-L1 molecular weight (Da)	33,275	https://www.phosphosite.org/proteinAction.action?id=19198&showAllSites=true
TMDD parameters—QE		
Target location	Plasma, lymph & interstitial	EMA Assessment Report (EMA, 2021)
R_max_ option	Dynamic	Burr et al. (2017)
PD-L1 K_D_ (µM)	0.000299	EMA Assessment Report (EMA, 2021)
PD-L1 K_int_ (1/hr)	0.606	Burr et al. (2017)

### Atezolizumab model verification in adults

To verify the model in adults, the simulated atezolizumab plasma concentration-time profiles following single dosing over a 667-fold dose range (0.03 mg/kg to 20 mg/kg) in adult patients with solid tumors and hematologic malignancies were compared to observed data (Herbst et al., 2014). The clinical data showed that atezolizumab exposure becomes dose proportional (i.e., linear PK) at dose levels >1 mg/kg (Herbst et al., 2014). Therefore, TMDD was expected to account for only a minor contribution to the overall clearance of atezolizumab at the relevant 15 mg/kg dose in pediatric patients. To confirm this, simulations were performed in adults and pediatrics receiving 15 mg/kg atezolizumab, including and excluding the TMDD component. Comparable PK was observed (Supplementary Figure S2), therefore TMDD was not included in the subsequent modeling and simulations. The PBPK model without the TMDD component was further verified by comparing the simulated atezolizumab exposure metrics in adult patients with observed data following a single dose of 1,200 mg and simulated data at steady state from the published population pharmacokinetic (PopPK) model following multiple dosing of 1,200 mg Q3W (Stroh et al., 2017) (Supplementary Table S3).

### Atezolizumab physiologically-based pharmacokinetic model development in pediatric patients

After the atezolizumab PBPK model for adults was developed and verified, age-dependent physiological and biochemical changes in tissue volumes (vascular, endosomal, interstitial, and intracellular), blood and lymph flows, hematocrit, and endogenous IgG concentration were incorporated as described previously (Johnson et al., 2006; Johnson and Rostami-Hodjegan, 2011; Pan et al., 2020) to establish the pediatric atezolizumab PBPK model. Notably, the ontogeny of endogenous IgG concentration was described by combining maternal and pediatric contributions using an exponential decline for maternal IgG and a saturable function for the increasing pediatric contribution (Pan et al., 2020). A quantitative measure of FcRn ontogeny is currently lacking in the literature, therefore the mean FcRn concentration in pediatric subjects was assumed the same as in adults. To capture interindividual variability, the FcRn concentration in each pediatric subject was calculated based upon the correlation with endogenous IgG (Li et al., 2014; Pan et al., 2020). When this correlation was enabled, the model predicted a reasonable variability in exogenous IgG t_1/2_ and captured the trajectory of exogenous IgG t_1/2_ in pediatric subjects in relation to endogenous IgG level (Pan et al., 2020). The ontogeny data of lymph flow were not available. Therefore, adult lymph flow was scaled allometrically with an exponent of 0.75 for pediatric subjects, resulting in a total lymph flow that was 2.6-fold higher in neonates than in adults (Pan et al., 2020). The percentage of total lymph flow coming from each specific organ/tissue was assumed the same in children as in adults (Gill et al., 2016). The potential changes of CL_cat_ and CL_add_ during pediatric growth was assumed to follow body weight-based allometric scaling with an exponent of 0.808, which was determined from a PopPK model (Stroh et al., 2017). This value was similar to the allometric exponent (0.81) published from an analysis of data for 23 mAbs in humans and cynomolgus monkeys (Betts et al., 2018). Since the ontogeny of PD-L1 in children was unknown and the high atezolizumab concentration after a dose of 15 mg/kg likely fully saturates the target, the model without TMDD was used for pediatric simulation and extrapolation. To test the validity of the exclusion of TMDD, simulations were performed in both adult and pediatric populations receiving 15 mg/kg atezolizumab, both including and excluding the TMDD model, and the predicted atezolizumab exposure was not significantly affected (Supplementary Figure S2).

### Individualized model verification in pediatric patients

To verify the model in pediatrics, the physiology of the simulated virtual pediatric subjects was redefined at regular intervals over the length of simulation based on the age, as the physiology of virtual pediatric subjects developed significantly during the simulation time. The redefining schedule was every 2 weeks for 2- to 6-year-old subjects and every month for >6-year-old subjects. These schedules were selected in order to seamlessly predict PK concurrent with subject maturation and resulted in a discrepancy <2% in all relevant physiological parameters (Abduljalil et al., 2014). An individualized model verification approach was used to evaluate the atezolizumab model in both pediatric patients and in adult patients. The simulated plasma atezolizumab concentrations for each virtual subject matching the enrolled patient’s unique age, sex, and dose regimen were compared with observed sparse sampling data obtained following multiple dosing (Q3W) of atezolizumab 15 mg/kg in 2- to 18-year-old pediatric patients and 1,200 mg in 18- to 29-year-old adult patients for up to 672 days (Shemesh et al., 2019). The simulated body weight distribution from virtual subjects was also compared to the actual body weight of enrolled patients because of the 15 mg/kg body weight-based dosing (Supplementary Figure S3).

### Physiologically-based pharmacokinetic model applications

Simulations until steady state were produced using the verified PBPK model for broadly representative virtual subjects from 2 to 18 years old after 15 mg/kg (not exceeding 1,200 mg) and subjects ≥ 18 years old after 1,200 mg IV atezolizumab. The age range of interest (2 to <18 yo) was stratified into 3 age bands: 2 to <6 yo, 6 to <12 yo, and 12 to <18 yo. Ten virtual trials of 20 pediatric subjects (50% female) for each age band was used as the simulation set-up. The values, statistics, and distributions of C_min_, C_max_, and AUC_tau_ after the first (cycle 1) and steady state (cycle 10) dose were derived.

### Sensitivity analysis

A sensitivity analysis was performed to evaluate the sensitivity of the simulated atezolizumab exposure to the individual clearance pathways implemented in the PBPK model. Atezolizumab exposure was simulated (C_min_, C_max_, and AUC_tau_) following a single dose and at steady state (15 mg/kg Q3W in children and 1,200 mg Q3W in adults) in 2- to 4-, 4- to 8-, 8- to 12-, and 12- to 18-year-old pediatric patients and in adult patients where only CL_cat_ or CL_add_ was included in the PBPK model.

Additionally, the impact of key parameters (K_up_, K_rc_, and FcRn abundance) on plasma atezolizumab concentration profile and systemic AUC was tested individually with other model parameters kept constant. The tested parameter range of K_up_, K_rc_, and FcRn abundance was 0.000298–2.98 h^−1^, 0.0313–313 h^−1^, and 0.1–1,000 μM, respectively.

## Results

### Physiologically-based pharmacokinetic model development and verification in adults

**FIGURE 3:**
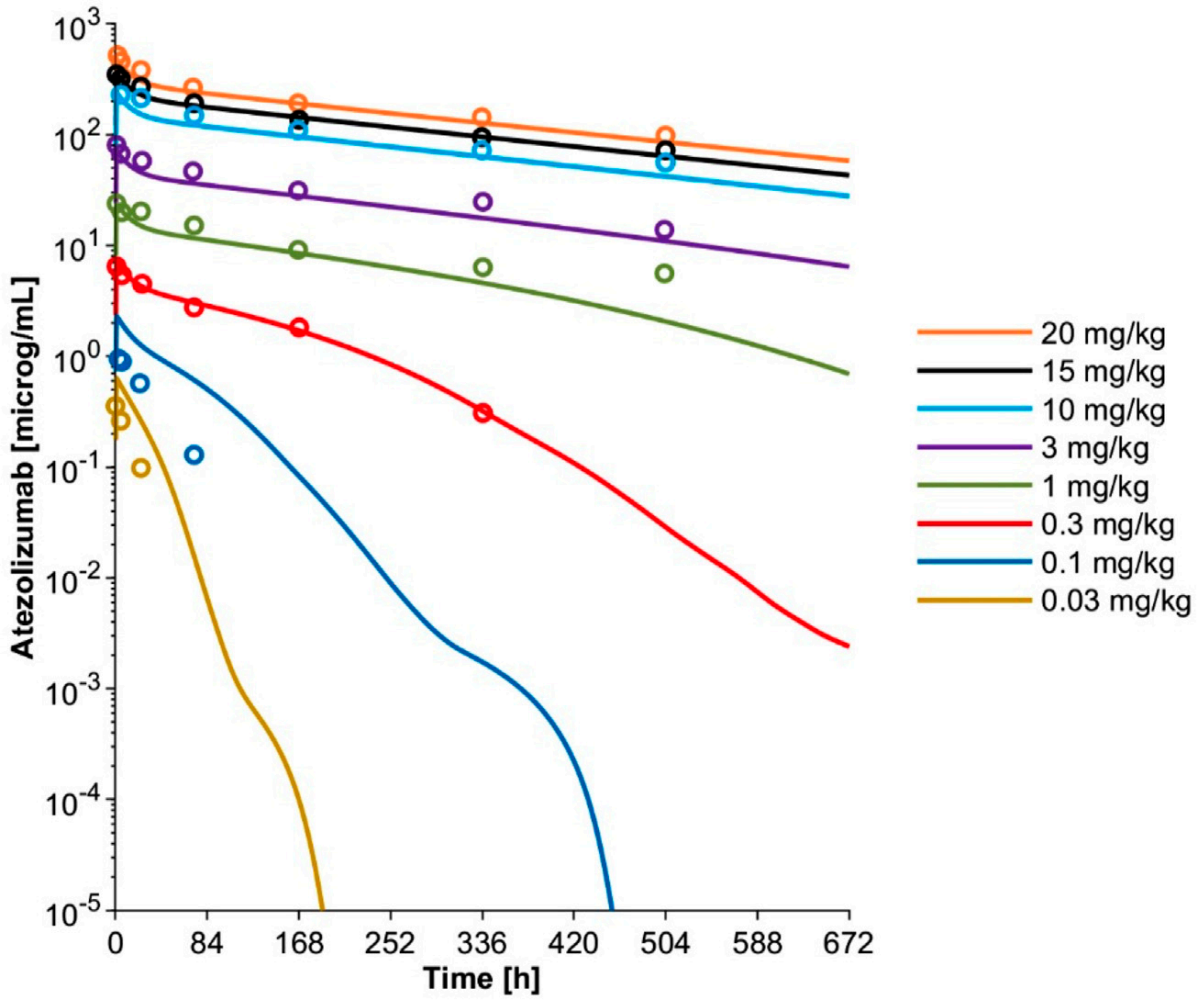
Simulated mean plasma concentration-time profiles and observed mean concentration data for adult patients with solid tumors and hematologic malignancies after a single IV dose at varying dose levels using the optimized final PBPK model including TMDD. Observed mean data are shown as open circles (Herbst et al., 2014). Simulated mean data are shown as solid lines.

The initial model with only lysosome based CL_cat_ overpredicted atezolizumab exposure compared to the clinical data after treatment with 20 mg/kg (Supplementary Figure S1A) (Herbst et al., 2014). Thus, CL_add_ was included and optimized as 0.00693 L/h to improve model performance and recapitulate the clinically observed data (Supplementary Figure S1B). The model with both CL_cat_ and CL_add_ overpredicted drug exposure at lower dose levels (Supplementary Figure S1C), necessitating the inclusion of a TMDD component, where PD-L1 target expression (R_max_) was optimized as 0.609 nM to recover the clinically observed data after receiving 0.3 mg/kg (Supplementary Figure S1D). The final TMDD model was then verified over a dose range of 0.03 to 20 mg/kg, where the atezolizumab concentration-time profiles at all dose levels were adequately captured (Figure 3). This confirmed that the minimal PBPK model with TMDD was able to successfully describe atezolizumab disposition in adults.

The TMDD pathway was excluded for pediatric patient modeling due to the minimal contribution of TMDD to atezolizumab disposition at the 15 mg/kg dose level and the lack of ontogeny data on the PD-L1 target. Simulations including and excluding the TMDD component were performed in adult and pediatric patients receiving a 15 mg/kg dose of atezolizumab to confirm that the exclusion of TMDD had minimal impact. These results showed that the predicted plasma atezolizumab concentration-time profile was not significantly different when TMDD was included or excluded (Supplementary Figure S2). The PBPK model without the TMDD component was further verified by comparing the simulated atezolizumab exposure metrics for adult patients with observed data following a single dose of 1,200 mg from a separate dataset (Stroh et al., 2017) as well as simulated data at steady state from a published PopPK model following multiple dosing of 1,200 mg Q3W (Stroh et al., 2017) (Supplementary Table S3). This comparison revealed that the simulated atezolizumab exposure metrics including C_min_, C_max_, and AUC_tau_ from the PBPK model without TMDD all agreed with both observed data and the PopPK model-simulated data after both single dosing and multiple dosing at cycle 1 and after cycle 10. Taken together, we showed that the TMDD pathway did not play a significant role in atezolizumab disposition at therapeutic dose levels; the minimal PBPK model without TMDD was successfully verified to capture atezolizumab PK after IV administration of 15 mg/kg in adults, which was considered for the subsequent modeling in pediatrics.

### Physiologically-based pharmacokinetic model development and individualized verification in pediatrics

**FIGURE 4:**
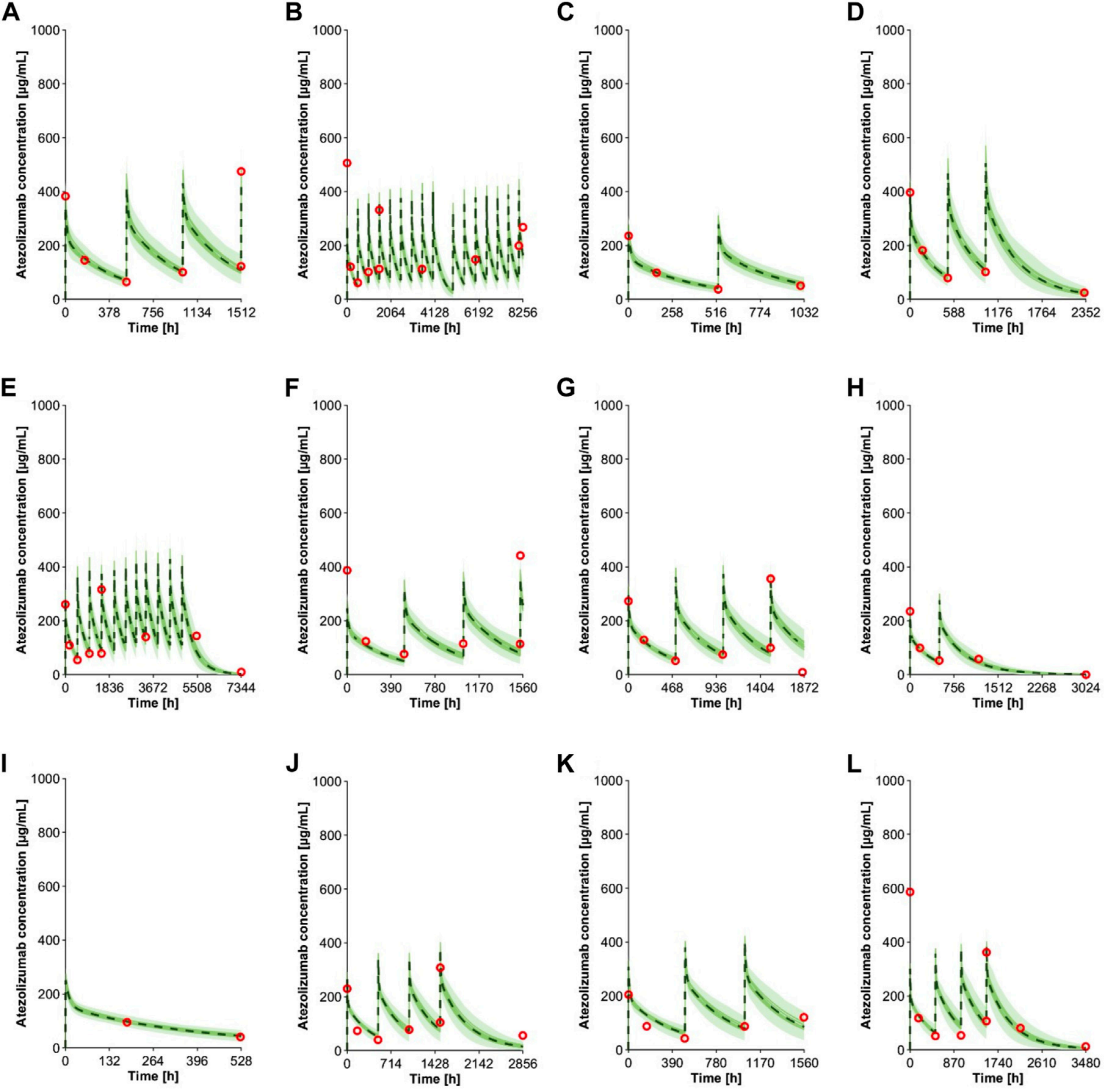
Individual observed plasma concentrations (Shemesh et al., 2019) and simulations of multiple IV doses of 15 mg/kg (<18 yo) or 1,200 mg (≥18 yo) atezolizumab Q3W in pediatrics. The panels show data from subjects with different age **(A)** 16 yo, **(B)** 15 yo, **(C)** 13 yo, **(D)** 12 yo, **(E)** 11 yo, **(F)** 10 yo, **(G)** 8 yo, **(H)** 7 yo, **(I)** 5 yo, **(J)** 4 yo, **(K)** 3 yo, and **(L)** 2 yo. Observed data (Shemesh et al., 2019) are shown in red open circles. The green lines represent simulated trials, and the dashed black lines represent the mean data for the entire simulated population (*n* = 100). The green shaded area represents the 5th to 95th percentiles of the simulations.

**FIGURE 5:**
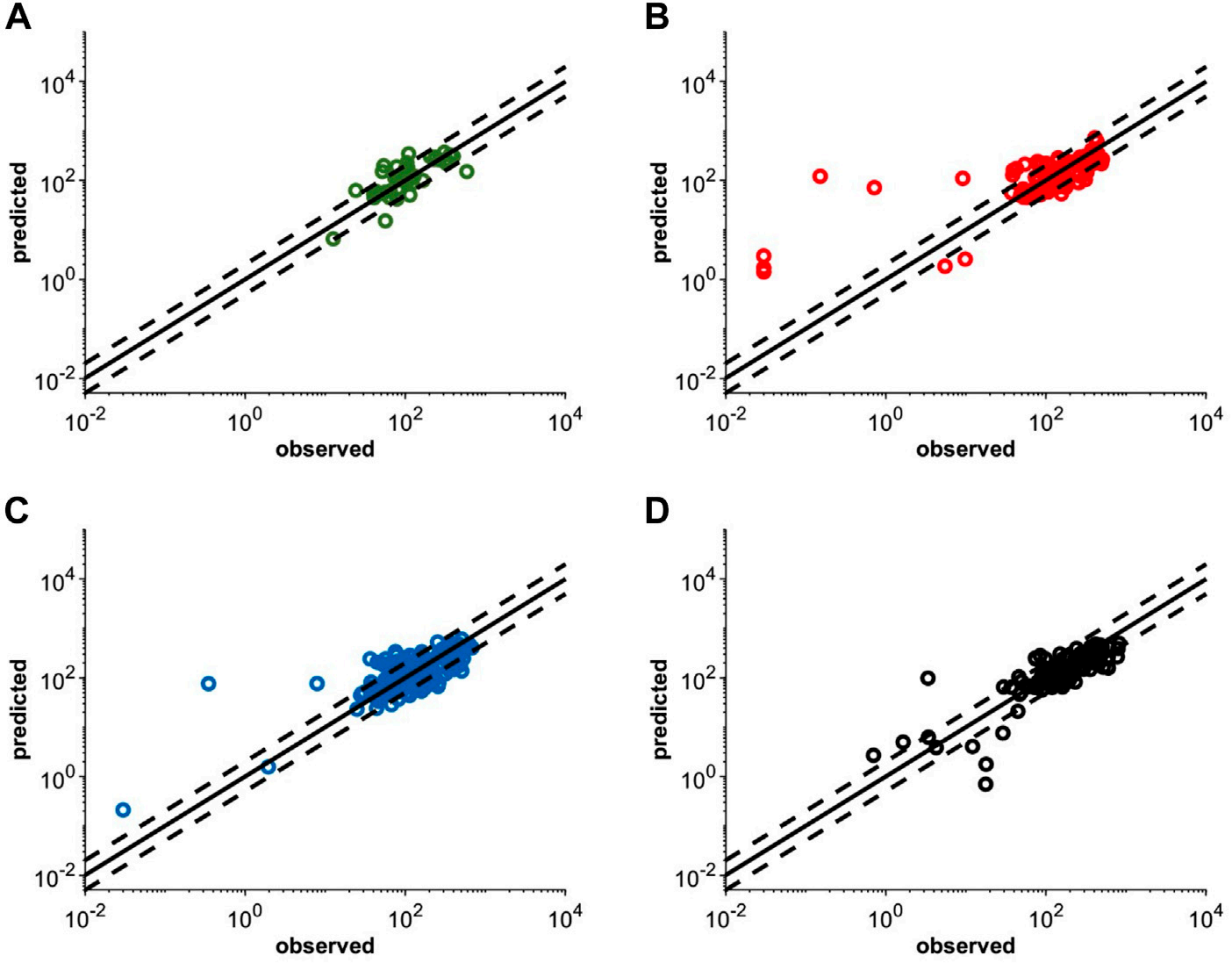
Individualized simulated mean and observed (Shemesh et al., 2019) plasma concentrations following multiple IV doses of 15 mg/kg (<18 yo) or 1,200 mg (≥18 yo) atezolizumab Q3W in pediatric patients **(A)** 0–6 yo, **(B)** 6–12 yo, and **(C)** 12–18 yo or **(D)** young adult patients with solid tumors and hematologic malignancies. The solid black lines represent the line of unity and the dashed black lines represent the 2-fold error margin. Each simulation matched the dosing information and demographics with each enrolled subject.

The virtual pediatric population in the Simcyp V20 was selected for pediatric modeling after the atezolizumab PBPK model was developed and verified for adults. An individualized model verification approach was used to evaluate the atezolizumab model for both pediatric subjects and young adults using individual observed data from every single enrolled subject (Shemesh et al., 2019). The virtual subjects in the model were matched to have the same age and gender as enrolled patients with comparable body weight (Supplementary Figure S3). The simulated concentration-time profiles adequately recovered the observed longitudinal data following multiple IV doses of atezolizumab (15 mg/kg in <18-year-olds and 1,200 mg ≥ 18-year-olds Q3W; representative data shown in Figure 4). A comparison of all simulated and observed plasma atezolizumab concentrations (N = 431) from 87 subjects is shown in Figure 5, stratified by age group (0–6 years, 6–12 years, 12–18 years, and 18–29 years). Overall, the simulated concentrations of atezolizumab were comparable (76% within two-fold and 90% within three-fold) to the observed data (Supplementary Table S4). There was no bias for either over- or underprediction, and prediction accuracy was similar across the age range (Supplementary Table S4; Figure 5). Notably, the accuracy was relatively reduced at the lower concentrations, which represented samples taken a long time after the final dose (Figure 5). Overall, the model performance indicates successful verification of the pediatric atezolizumab PBPK model.

### Physiologically-based pharmacokinetic model applications

**FIGURE 6:**
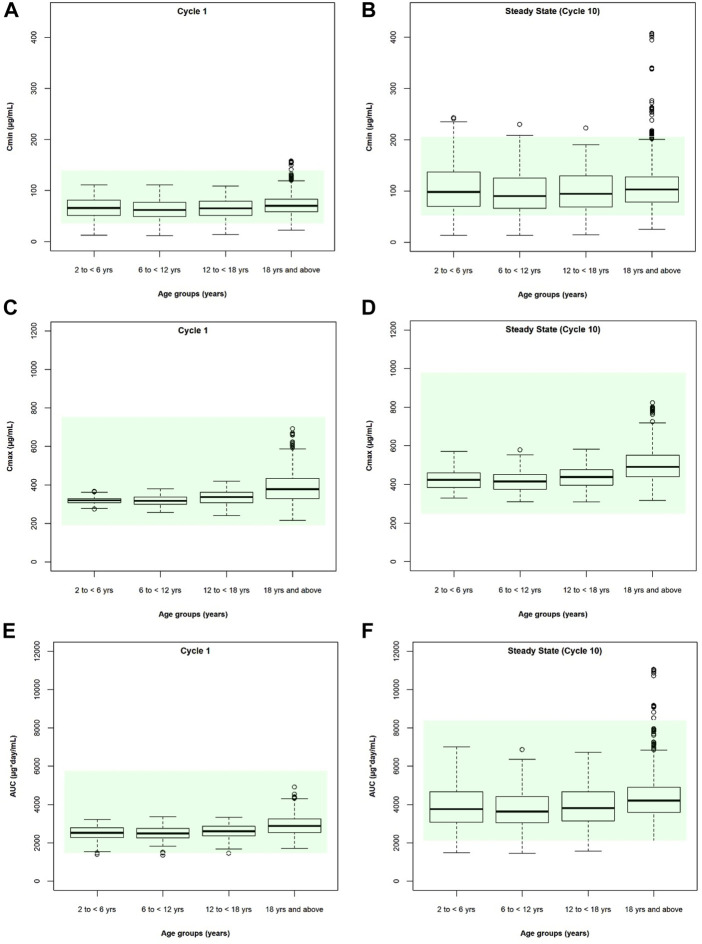
Cycle 1 and steady state (cycle 10) simulated atezolizumab **(A,B)** C_min_, **(C,D)** C_max_, and **(E,F)** AUC_tau_ for pediatric (2-6 yo, 6–12 yo, and 12–18 yo) and adult (≥18 yo) patients following multiple IV doses of 15 mg/kg (<18 yo) or 1,200 mg (≥18 yo) atezolizumab Q3W. The box represents the median value and interquartile range; error bars represent the 5th and 95th percentiles. Circles represent individual subjects outside of the 5th to 95th percentiles. Shaded area represents the simulated adult acceptable criteria (50%–200% of the adult median value).

After individualized verification for pediatric patients, the model was applied to produce simulations until steady state in a representative virtual population. The distribution of exposure metrics including C_min_, C_max_, and AUC_tau_ were not significantly different between children and adults (Figure 6), with a geometric mean of pediatric exposure metrics between 0.8- to 1.25-fold of adult value (Table 2), although slightly lower C_min_, C_max_, and AUC_tau_ were observed in pediatric subjects. At both Cycle 1 and at steady state (Cycle 10), no pediatric subjects were predicted to have a C_max_ outside of the acceptable adult criteria (50%–200% of the adult median value) (Figure 6; Supplementary Table S5). For Cycle 1, C_min_ values were predicted to be <50% of the adult median value in 11.5%, 11.0%, and 11.5% of subjects aged 2- to 6-year-old, 6- to 12-years old, and 12- to 18-year-old, respectively; no subjects were predicted to be >200% adult median value in any age group. At steady state (Cycle 10), C_min_ values were predicted to be <50% of the adult median value in 10.5%, 10.0%, and 10.5% of subjects and >200% adult median value in 4.0%, 1.0%, and 0.5% of subjects aged 2- to 6-year-old, 6- to 12-years old, and 12- to 18-year-old, respectively (Supplementary Table S6). No pediatric subjects were predicted to have a Cycle 1 C_min_ <6 μg/ml, which was the minimal effective concentration (Deng et al., 2016). For Cycle 1, AUC_tau_ values were predicted to be <50% of the adult median value in 0.5% of subjects aged 2- to 6-year-old and 6- to 12-year-old, and no subjects aged 12- to 18-year-old. At steady state, AUC_tau_ values were predicted to be <50% of the adult median value in 1.5%, 1.5%, and 2.5% of subjects aged 2- to 6-year-old, 6- to 12-year-old, and 12- to 18-year-old, respectively (Supplementary Table S7). No pediatric subjects were predicted to have an AUC_tau_ greater than the adult acceptable criteria (200% of the adult median value) following a single dose or at steady state. Overall, similar atezolizumab exposure was observed in pediatric patients regardless of age when compared with adults at both Cycle 1 and at steady state.

**TABLE 2:** Predicted geometric mean plasma C_min_, C_max_, and AUC_tau_ values for atezolizumab following multiple IV doses of 15 mg/kg atezolizumab Q3W in pediatric subjects and 1,200 mg in adults for Cycle 1 and Cycle 10 (steady state). Relative values of pediatric exposure metrics compared to adults are also shown.

Cycle		C_min_ (μg/ml)	C_max_ (μg/ml)	AUC_tau_ (μg × day/ml)	C_min_	C_max_	AUC_tau_
					Relative to adult		
1	≥18 yo (adult)	68.2	380	2,894	1.00	1.00	1.00
	2 to 6 yo	62.2	319	2,483	0.91	0.84	0.86
	6 to 12 yo	59.3	316	2,464	0.87	0.83	0.85
	12 to 18 yo	60.8	333	2,557	0.89	0.88	0.88
10	≥18 yo (adult)	100	492	4,187	1.00	1.00	1.00
	2 to 6 yo	93.4	423	3,737	0.94	0.86	0.89
	6 to 12 yo	87.7	414	3,612	0.88	0.84	0.86
	12 to 18 yo	89.3	434	3,730	0.90	0.88	0.89

### Sensitivity analysis

As the simulated atezolizumab exposure was relatively flat over the 2–18 year age range, a sensitivity analysis was performed to investigate the impact of each clearance pathway. When the PBPK model included only clearance *via* catabolism within the lysosomes (CL_cat_), exposure at steady state increased as age decreased; particularly, the impact is most pronounced for 2- to 12-yo children (Supplementary Figure S4). This reflects the impact of lower IgG levels in children, which results in reduced competition for binding to FcRn, more efficient recycling, less catabolism, and hence higher drug exposure. In contrast, when the PBPK model included only clearance *via* unspecified additional clearance (CL_add_), exposure decreased as age decreased (Supplementary Figure S5), due to the allometric exponent of 0.808 (Stroh et al., 2017) being applied to CL_add_ within the model, leading to higher clearance per kg in younger children that typically have lower body weight. When considering CL_cat_ and CL_add_ together in the final PBPK model, the total clearance resulted in a flat exposure predicted over the 2–18 year age range.

In addition, the effect of each of the key optimized parameters (K_up_, K_rc_, and FcRn abundance) on plasma atezolizumab concentration profile and systemic AUC was tested. The current values of these parameters are within the sensitive ranges where simulation results may fluctuate significantly as the parameter value changes (Supplementary Figure S6). As K_up_ increases, systemic AUC decreases due to more endocytosis and subsequent catabolism. In contrast, as K_rc_ increases, systemic AUC increases due to more drug recycling and rescue from catabolism. Finally, as FcRn increases, the systemic AUC also increases due to increased binding to FcRn and protection from catabolism.

## Discussion

In this study, we developed a mechanistic PBPK model for atezolizumab (Figures 1, 2) using both *in vitro* and *in vivo* data and verified our model for adults (Figure 3; Table 1). We then incorporated maturation and verified the model for pediatric patients using individual clinically observed data from 87 children and young adults aged from 0 to 29 years old (Figures 4, 5). The verification showed unequivocally that our model was able to recapitulate atezolizumab disposition in both adult and pediatric populations. The verified model was then applied to generate simulations in the broadly representative virtual populations across all age groups. The results indicated that atezolizumab exposure was comparable between children from 2- to 18-year-old following IV administration of 15 mg/kg (max 1,200 mg) atezolizumab Q3W and adults after 1,200 mg IV Q3W (Figure 6; Table 2), supporting our 15 mg/kg dose recommendation for atezolizumab in pediatric patients.

To date, pediatric studies are required when new molecular entities are being developed unless waived or deferred (Johnson and Ke, 2021). It is critical to understand the efficacy, safety, tolerability, and PK of drugs in children for appropriate and optimal drug utilization. However, blood samples may only be sporadically collected from pediatric patients to study the PK in this population because of the burden to children, blood sample volume limitation, and other logistical challenges (Abdel-Rahman et al., 2007; Joseph et al., 2015). Thus, there exists a need for the use of PK modeling and simulation techniques to understand and predict PK in children. This can facilitate safe and effective first-in-child dose design, supplement limited exposure data in children and support pediatric dosing recommendations.

Generally, dose recommendations in pediatric patients leverage empirical allometric scaling or mechanistic PBPK modeling (Germovsek et al., 2021; Johnson and Ke, 2021). The former approach uses power functions to calculate the drug clearance or volume of distribution based on the normalized body weight or body surface area of a pediatric subject with respect to an adult. In contrast, mechanistic PBPK models represent the biological system and consider age-dependent changes of physiology, anatomy, and biochemistry that together govern the drug disposition in the human body (Edginton et al., 2006; Johnson et al., 2006). The PBPK model provides a unique advantage over allometric scaling because all physiologically important components and pathways are integrated within one framework in an inter-dependent and non-linear fashion. Furthermore, the maturation effect on drug disposition is derived as an aggregate of changes in each individual mechanism, which better represents the reality and complexity of the human system and system-drug interactions.

Over the past decade, the application of PBPK modeling in pediatric extrapolation has gained critical mass, particularly in small molecules (Huang et al., 2017; Lutz et al., 2021; Sinha et al., 2021; Zhu et al., 2021; Johnson et al., 2022), and regulatory agencies have shown general endorsement of such application (Luzon et al., 2017; Grimstein et al., 2019; Yellepeddi et al., 2019; Johnson and Ke, 2021). The rise of model-informed drug development initiatives provides a regulatory pathway for engaging pharmaceutical companies with the FDA, which allows for the incorporation of scientific findings and discussions into pediatric drug development with better transparency, alignment, and efficiency (Madabushi et al., 2019; Wang et al., 2019; Zhu et al., 2019).

However, the use of PBPK modeling in pediatrics is reported much less for mAbs when compared with small molecules. Thus, the applicability of the PBPK model for mAbs has not been extensively evaluated. One reason for this could be that mAbs represent a relatively newer modality and the number of marketed mAbs is considerably less than the number of small molecule drugs. Moreover, the mechanism of distribution and clearance differ dramatically between mAbs and small molecules, so previous knowledge of small molecules (e.g., the ontogeny profile of drug metabolizing enzymes) may not be relevant or applicable to mAbs. A significant knowledge gap of ontogeny exists in mechanisms specifically influencing the PK of mAbs such as transcytosis, FcRn expression, and lysosome-mediated degradation (Malik and Edginton, 2018; Malik and Edginton, 2020; Temrikar et al., 2020), where assumptions on the maturation and calibration using clinical data are needed to perform PBPK modeling and simulation. In spite of these challenges, six pediatric PBPK studies were recently conducted for more than ten biologics using either full-body or minimal structure using Simcyp or PK-Sim platforms (Hardiansyah and Ng, 2018; Hanke et al., 2019; Malik and Edginton, 2019; Basu et al., 2020; Malik and Edginton, 2020; Pan et al., 2020). Although these studies showed acceptable model performance, inconsistent approaches were utilized in order to capture PK of different drugs. To date, no consensus has been reached regarding the best methodology for PBPK modeling in pediatric patients for biologics.

In this study, our results indicated that atezolizumab exposure was comparable in subjects from 2 to 18 years old following IV administration of 15 mg/kg (max 1,200 mg) atezolizumab (Figure 6; Table 2). The reason for the flat exposure independent of age was mainly due to the cancellation of maturation effects on two separate clearance pathways: catabolism and unspecified additional clearance. The atezolizumab PBPK model considered changes in IgG concentrations and competitive binding to FcRn. Following birth, the IgG levels in children first drop as maternal IgG is eliminated and reach a nadir concentration around month three to four, after which IgG concentrations start to increase due to *de novo* synthesis of their own IgG, gradually reaching a plateau at the age of 8–10 years (Allansmith et al., 1968; Aksu et al., 2006; Pan et al., 2020). This results in less competition for FcRn binding and more efficient recycling of mAbs, which leads to diminished drug catabolism in younger children when IgG levels are low. On the other hand, the unspecified additional clearance (CL_add_) was scaled with a PopPK model-estimated exponent of 0.808 on body weight (Stroh et al., 2017) causing a higher CL_add_ per kg in younger children. Our sensitivity analyses show that the atezolizumab exposure in children would have been higher or lower if only CL_cat_ or CL_add_ was included in the model, respectively (Supplementary Figures S4, S5).

This study has several limitations. Quantitative measure of the ontogeny profile for lymph flow and FcRn are lacking in humans. To account for the real-time growth, the changes in lymph flow in the pediatric PBPK model were based on an allometric approach to be consistent with data reported in pre-clinical species (Pan et al., 2020). For FcRn, the concentration was assumed to be the same between children and adults. However, a correlation method was used between individual FcRn and endogenous IgG concentration as described before (Pan et al., 2020), which successfully captured the changes in exogenous IgG terminal half-life when administered to children of different ages. The model does not account for target binding in pediatric patients and the ontogeny of PD-L1 in children is unknown. For this reason, the model is only suitable for prediction of therapeutic dose levels in pediatric patients, where TMDD is saturated and only has minimal impact on PK. In addition, development of anti-drug antibody (ADA) was not considered in the current model. Based on findings from iMATRIX study (Geoerger et al., 2020), the observed ADA incidence rate in pediatrics was 15% (9/60) which was less than or similar to that of the typical ADA incidence in adults in various indications (Shemesh et al., 2019). Lastly, we implemented an additional clearance (CL_add_) as an unspecified pathway for unknown mechanisms, which did not incorporate any ontogeny and might not be accurately captured in children. Because the CL_add_ was optimized empirically and minimal PBPK model was used, there is a potential risk that this parameter was overleveraged, which may be mitigated upon obtaining more mechanistic understanding of atezolizumab or applying a full body PBPK model. So far, the applicability of the Simcyp platform to biologics prediction in pediatrics has only been verified for six drugs and the confidence in 0- to 2-year-old population remains low (Pan et al., 2020). For atezolizumab, PK data from 0- to 2-year-old population were only available in two subjects (Shemesh et al., 2019). More modeling and simulation studies along with more pediatric PK data are necessary to obtain higher confidence in the model applicability. As we acquire more knowledge, the PBPK model framework will be updated as an iterative process together with more pediatric data and simulation case studies.

In conclusion, the application of mAb PBPK model offers much promise, and this work allowed us to understand the atezolizumab exposure at the dose level of 15 mg/kg in pediatric patients from 2- to 18-year-old with solid tumors in relation to adults. These findings support the optimal and safe use of atezolizumab at this dose level for label recommendation.

## Data Availability

The original contributions presented in the study are included in the article/Supplementary Material, further inquiries can be directed to the corresponding author.
