# Supplementary material for: Development of a pediatric physiologically-based pharmacokinetic model to support recommended dosing of atezolizumab in children with solid tumors
Source: Front Pharmacol. 2022 Sep 26;13:974423. doi: 10.3389/fphar.2022.974423 (PMC9548535; doi:10.3389/fphar.2022.974423)
Supplement: Supplementary file 1 [file DataSheet1.PDF]

Supplementary Material for

Development of a Pediatric Physiologically-based Pharmacokinetic Model to Support Recommended Dosing of Atezolizumab in Children with Solid Tumors

Weize Huang[1*], Felix Stader[2], Phyllis Chan[1], Colby S. Shemesh[1], Yuan Chen[1], Katherine L. Gill[2], Hannah M. Jones[2], Linzhong Li[3], Gianluca Rossato[4], Benjamin Wu[1], Jin Y. Jin[1], Pascal Chanu[1]

[1]Genentech Inc., South San Francisco, CA

[2]Certara UK Limited, Sheffield, UK

[3]Daiichi Sankyo, Inc., Basking Ridge, NJ (Current)

[4]F. Hoffmann-La Roche Ltd, Basel, Switzerland

[*]Corresponding author

Corresponding Author: Weize Huang, 1-DNA Way, South San Francisco, CA 94080-4990, huang.weize@gene.com

Key Words: alveolar soft part sarcoma, atezolizumab, physiologically-based pharmacokinetic (PBPK) modeling, pediatric extrapolation, pediatric oncology

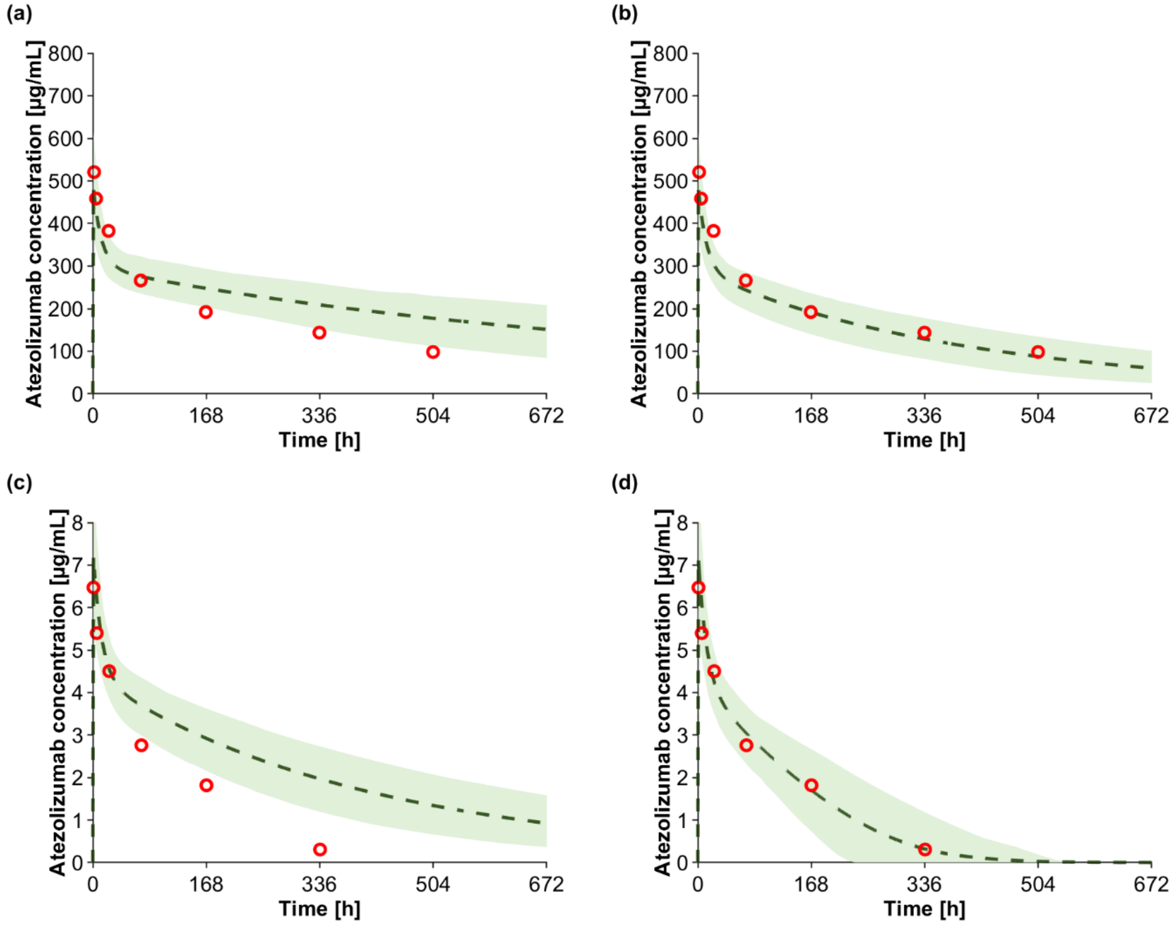

Figure S1. Simulated and observed mean plasma concentration-time profiles in adult patients with solid tumors and hematologic malignancies, after a single IV dose of (A) 20 mg/kg atezolizumab with no CL_{add} and no TMDD; (B) 20 mg/kg atezolizumab with optimized CL_{add} and no TMDD; (C) 0.3 mg/kg atezolizumab with optimized CL_{add} and no TMDD; (D) 0.3 mg/kg atezolizumab with optimized CL_{add} and optimized TMDD. Observed mean data are shown in red open circles (Herbst et al., 2014). The green lines represent simulated individual trials and the dashed black line the mean data for the simulated population (n = 100). The green shaded area represents the 5th to 95th percentiles.

Table S1. *In vitro* PD-L1 K_{deg} values including the median PD-L1 K_{deg} of 0.0426 1/h calculated from values presented in multiple publications.

Study	K_{deg} (1/h)	Cell line used
Burr et al., 2017	0.1422	Pancreatic cancer
Wang et al., 2019	2.1335	Human colorectal cancer
Yang et al., 2019	0.006	Breast cell cancer
Zhang et al., 2019	0.0196	Non-squamous cell lung cancer
Cha et al., 2018	0.0189	Human breast cell cancer
Yu et al., 2019	0.0656	Head and squamous cell cancer
Median	0.0426	-

Table S2. Atezolizumab K_D values for PD-L1 (EMA Assessment Report) including the weighted mean K_D value of 0.299 nM calculated from the reported in vitro values. Equilibrium binding studies to determine atezolizumab K_D for PD-L1 were performed using 3 lots of atezolizumab and its chimeric derivative PRO304397 with human PD-L1 expressed on 293 cells (EMA Assessment Report).

Atezolizumab Lot	K_D (nM)	Number of experiments
729339	0.433	1
	0.400	1
729341	0.228	3
602044	0.255	3
PRO304397	0.374	1
	0.336	1
Weighted mean	0.299	-

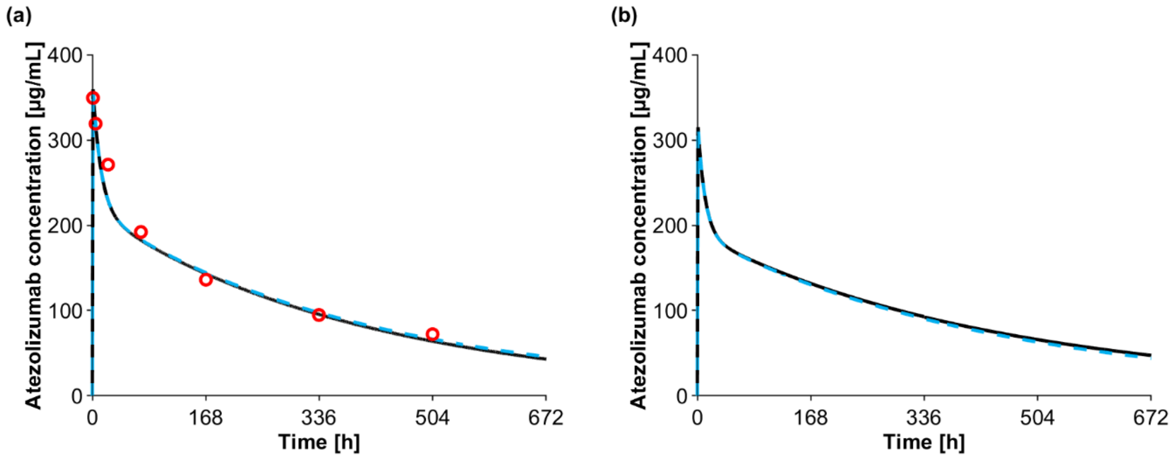

Figure S2. Observed PK data (Herbst et al., 2014, in red open circles) and simulated mean plasma concentration-time profiles (n=100) using the PBPK model including (black) and excluding (blue) TMDD after a single IV dose of 15 mg/kg atezolizumab in (A) adults, and (B) pediatrics (2-4 yo).

Table S3. Predicted and observed geometric mean exposure metrics for atezolizumab after the first cycle and ten cycles of IV 1200 mg atezolizumab Q3W for in patients with solid tumors and hematologic malignancies.

	Cycle 1			Cycle 10		
	AUC_{tau} (µg.day/mL)	C_{max} (µg/mL)	C_{min} (µg/mL)	AUC_{tau} (µg.day/mL)	C_{max} (µg/mL)	C_{min} (µg/mL)
PBPK Simulated	2875	363	70.7	4287	481	106
CV%	19.1	21	34.1	29.3	18.7	52
5th Percentile	2186	274	40.4	2800	370	52.1
95th Percentile	3807	512	106	6355	663	198
Observed	2874	379	72.1	-	-	-
CV%	24.2	22.2	46.1	-	-	-
PBPK/Observed	1	0.96	0.98	-	-	-
POPPK Predicted	2986	388	75.9	5746	570	168
CV%	26.9	28.1	44.4	48.2	33	75.7
PBPK/POPPK	0.96	0.94	0.93	0.75	0.84	0.66

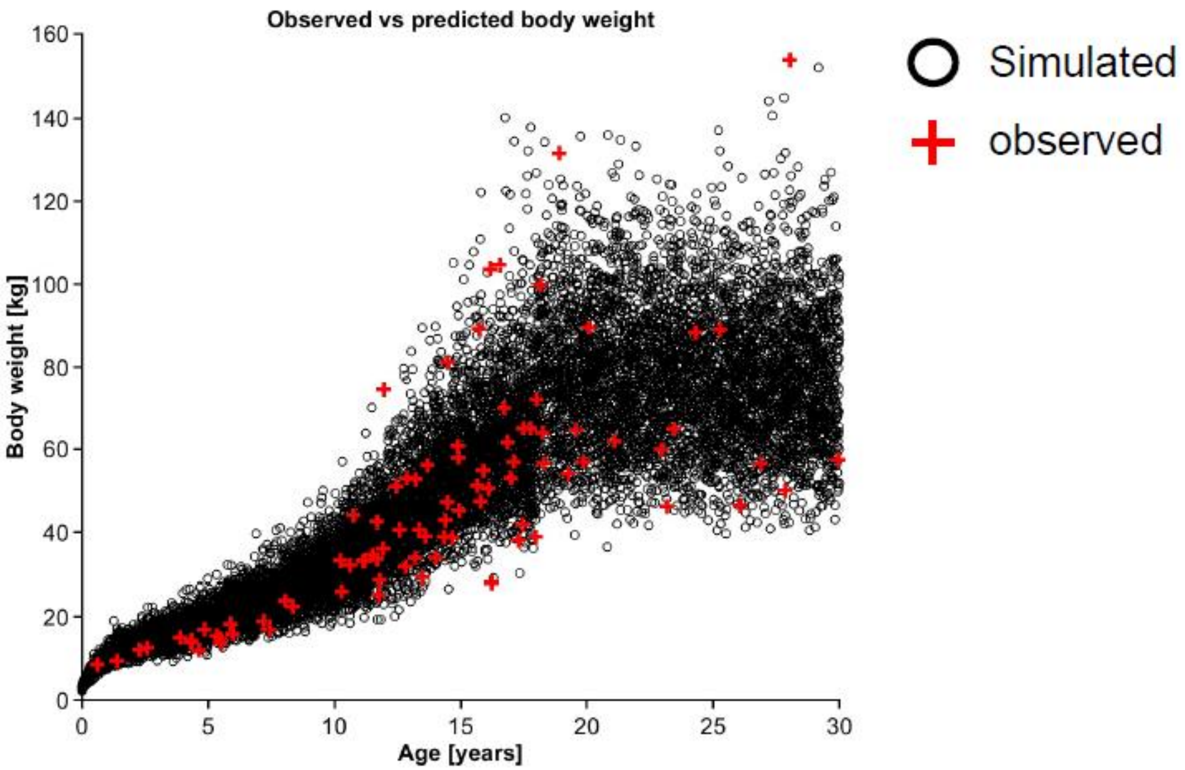

Figure S3. Simulated body weight of each virtual subject in comparison to observed body weight of enrolled subjects from age 0.6 to 29 yo.

Table S4. Predicted/observed concentrations for atezolizumab after multiple IV doses of 15 mg/kg (< 18 years) or 1200 mg (≥ 18 years) atezolizumab Q3W in pediatric and young adult patients with solid tumors and hematologic malignancies.

Predicted/Observed concentration ratios	% concentrations predicted within range	% concentrations underpredicted	% concentrations overpredicted
**1.25-fold**	39.5	21.6	17.9
**1.5-fold**	60.1	29.8	30.3
**2-fold**	76.4	34.3	42.2
**3-fold**	90.0	38.8	51.2
**5-fold**	95.9	42.3	53.5

Simulation uses Simcyp default IgG ontogeny; source observed data: Shemesh et al., 2019

Table S5. Predicted C_{max} values for atezolizumab following multiple IV doses of 15 mg/kg (not exceeding 1200 mg) atezolizumab Q3W for 10 cycles in adult and pediatric subjects.

**Cycle**	**1**				**10**			
**Age (y)**	**≥ 18 (adult)**	**2 to 6**	**6 to 12**	**12 to 18**	**≥ 18 (adult)**	**2 to 6**	**6 to 12**	**12 to 18**
**Geometric mean [CV%] (µg/mL)**	380 [21.9]	319 [5.54]	316 [7.46]	333 [10.4]	492 [18.0]	423 [13.3]	414 [13.4]	434 [14.3]
**Median (µg/mL)**	378	320	317	336	491	424	415	439
**5th – 95th percentile (µg/mL)**	279 - 523	290 - 347	282 - 352	286 - 387	373 - 643	346 - 519	336 - 502	347 - 528
**Geometric mean relative to adult**	1.00	0.84	0.83	0.88	1.00	0.86	0.84	0.88
**Median relative to adult**	1.00	0.85	0.84	0.89	1.00	0.86	0.85	0.89
**% < adult 5th percentile**	-	1.00	4.00	3.00	-	16.0	23.0	15.5
**% < 50% of adult median**	-	0.00	0.00	0.00	-	0.00	0.00	0.00
**% > adult 95th percentile**	-	0.00	0.00	0.00	-	0.00	0.00	0.00
**% > 200% of adult median**	-	0.00	0.00	0.00	-	0.00	0.00	0.00

-, Not applicable

Table S6. Predicted C_{min} values for atezolizumab following multiple IV doses of 15 mg/kg (not exceeding 1200 mg) atezolizumab Q3W for 10 cycles in adult and pediatric subjects.

**Cycle**	**1**				**10**			
**Age (y)**	**≥ 18 (adult)**	**2 to 6**	**6 to 12**	**12 to 18**	**≥ 18 (adult)**	**2 to 6**	**6 to 12**	**12 to 18**
**Geometric mean [CV%] (µg/mL)**	68.2 [34.3]	62.2 [44.9]	59.3 [42.1]	60.8 [43.2]	100 [51.4]	93.4 [67.9]	87.7 [61.1]	89.3 [63.2]
**Median (µg/mL)**	70.2	65.9	61.9	65.3	103	98.3	90.1	94.6
**5th – 95th percentile (µg/mL)**	38.3 - 103	30.7 - 99.1	33.1 - 93.5	30.3 - 94.9	46.2 - 190	35.9 - 197	40.2 - 173	35.4 - 173
**Geometric mean relative to adult**	1.00	0.91	0.87	0.89	1.00	0.94	0.88	0.90
**Median relative to adult**	1.00	0.94	0.88	0.93	1.00	0.95	0.87	0.92
**% < adult 5th percentile**	-	11.5	11.0	11.5	-	10.5	10.0	10.5
**% < 50% of adult median**	-	9.50	9.00	10.5	-	11.5	15.0	14.5
**% > adult 95th percentile**	-	4.00	1.00	0.5	-	6.50	2.50	1.00
**% > 200% of adult median**	-	0.00	0.00	0.00	-	4.00	1.00	0.50
**% < 6 µg/mL in cycle 1**	-	0.00	0.00	0.00	-	-	-	-

-, Not applicable

Table S7. Predicted AUC_{tau} values for atezolizumab following multiple IV doses of 15 mg/kg (not exceeding 1200 mg) atezolizumab Q3W for 10 cycles in adult and pediatric subjects.

**Cycle**	**1**				**10**			
**Age (y)**	**≥ 18 (adult)**	**2 to 6**	**6 to 12**	**12 to 18**	**≥ 18 (adult)**	**2 to 6**	**6 to 12**	**12 to 18**
**Geometric mean [CV%] (µg/mL)**	2894 [19.1]	2483 [16.4]	2464 [16.1]	2557 [16.4]	4187 [27.8]	3737 [35.2]	3612 [31.7]	3730 [32.0]
**Median (µg/mL)**	2889	2530	2496	2614	4209	3764	3640	3817
**5th – 95th percentile (µg/mL)**	2128 - 3846	1874 - 3044	1918 - 3034	1942 - 3125	2759 - 6020	2217 - 5966	2316 - 5496	2305 - 5590
**Geometric mean relative to adult**	1.00	0.86	0.85	0.88	1.00	0.89	0.86	0.89
**Median relative to adult**	1.00	0.88	0.86	0.90	1.00	0.89	0.86	0.91
**% < adult 5th percentile**	-	13.0	16.5	14.5	-	14.0	17.0	16.0
**% < 50% of adult median**	-	0.50	0.50	0.00	-	1.50	1.50	2.50
**% > adult 95th percentile**	-	0.00	0.00	0.00	-	5.00	1.00	0.50
**% > 200% of adult median**	-	0.00	0.00	0.00	-	0.00	0.00	0.00

-, Not applicable

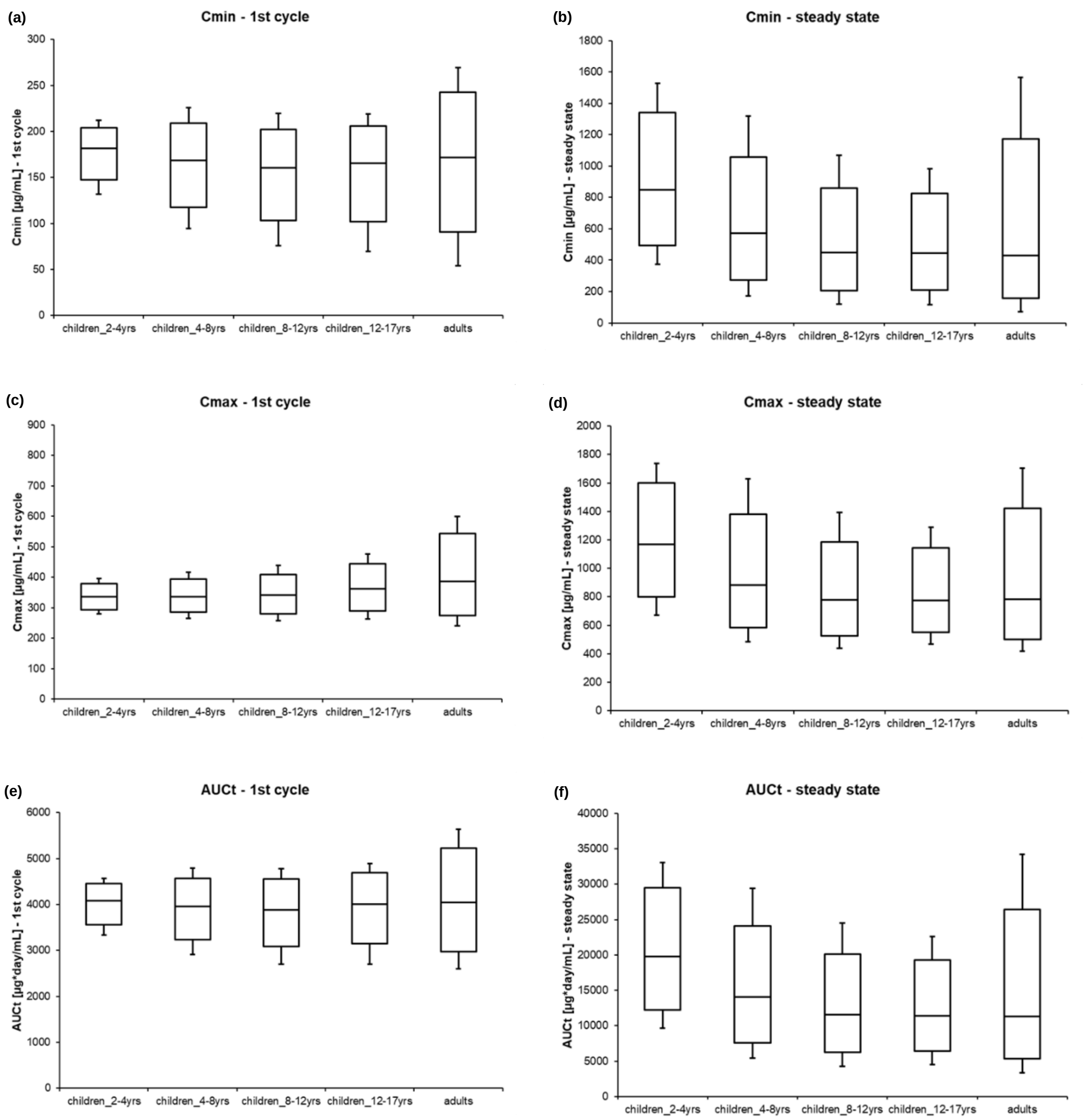

Figure S4. Simulated atezolizumab (A), (B) C_{min}; (C), (D) C_{max}; and, (E), (F) AUC_{tau} for adults and pediatric patients aged 2 to 18 years with solid tumors or hematologic malignancy when only CL_{cat} is included in the PBPK model. The box represents the median value, 5th and 95th percentiles; error bars represent the minimum and maximum value simulated for individuals aged 2-4 yr, 4-8 yr 8-12 yr, 12-18 yr and adults.

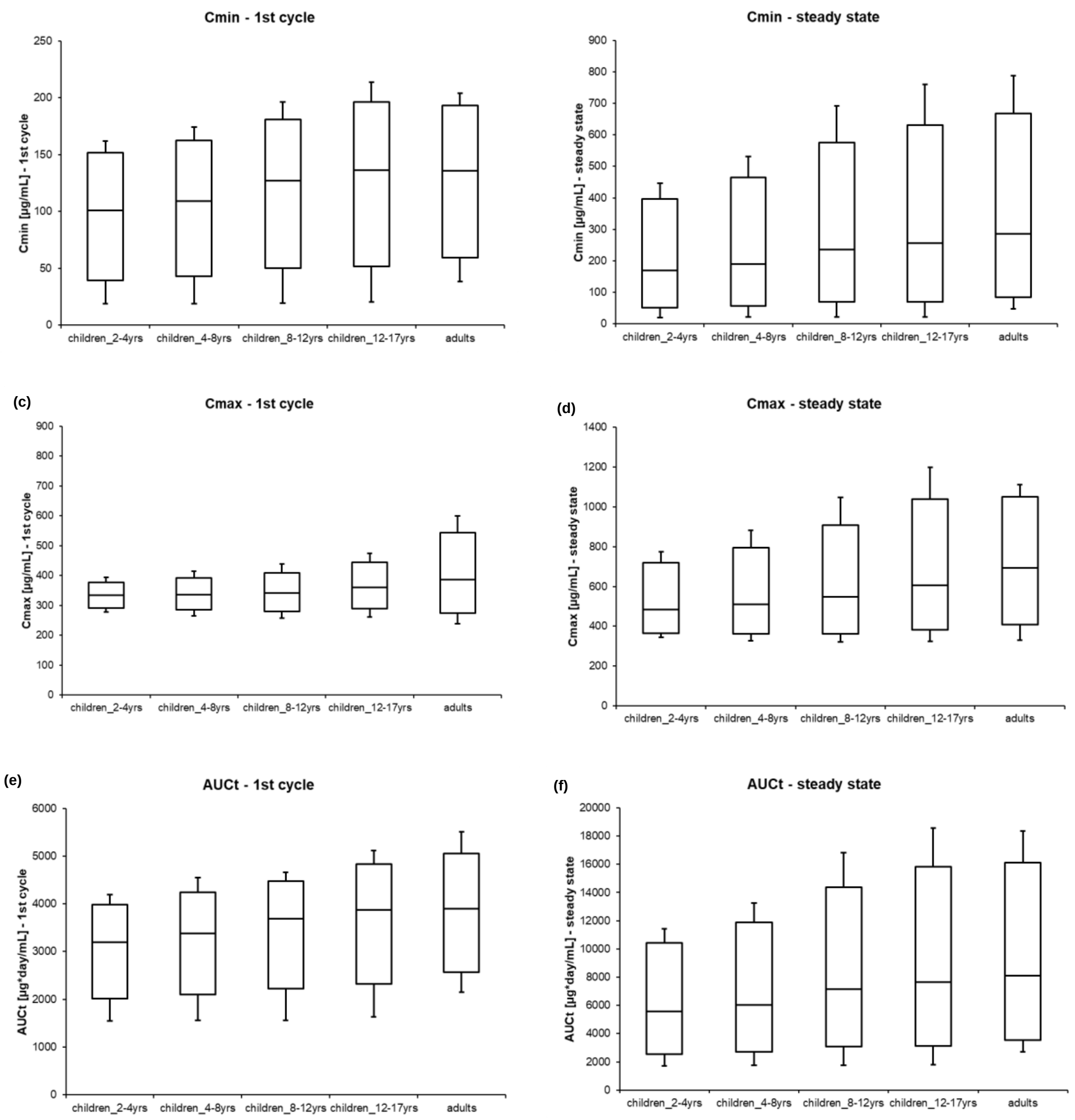

Figure S5. Simulated atezolizumab (A), (B) C_{min}; (C), (D) C_{max} ; and, (E), (F) AUC_{tau} for adults and pediatric patients aged 2 to 18 years with solid tumors or hematologic malignancy when only CL_{add} is included in the PBPK model. The box represents the median value, 5th and 95th percentiles; error bars represent the minimum and maximum value simulated for individuals aged 2-4 yr, 4-8 yr 8-12 yr, 12-18 yr and adults.

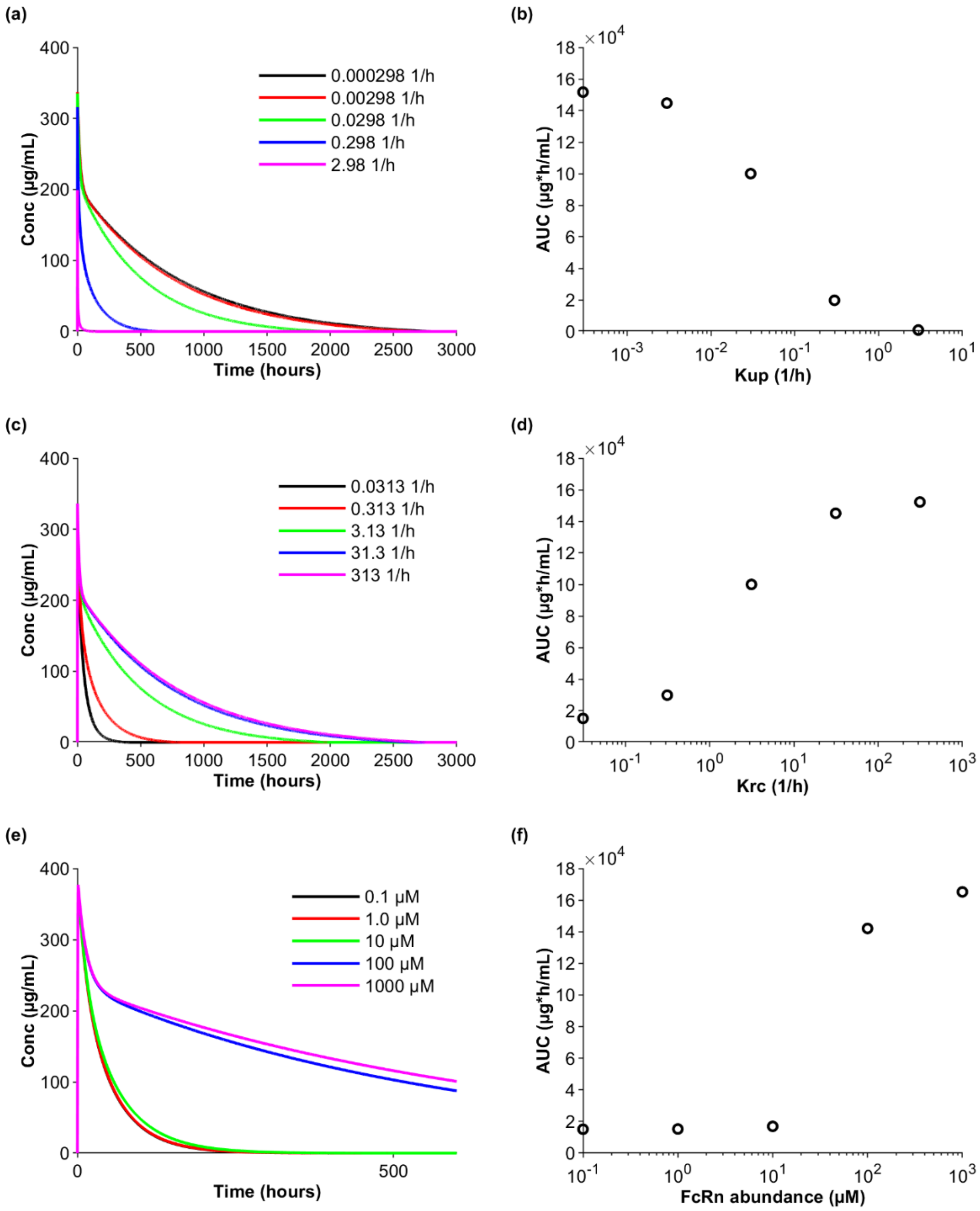

Figure S6. Sensitivity analysis of key parameters including K_{up} (panels A-B), K_{rc} (panels C-D), and FcRn abundance (panels E-F) on plasma atezolizumab concentration profile (left column) and systemic AUC (right column). The tested parameter range of K_{up}, K_{rc}, and FcRn abundance was 0.000298 – 2.98 hr-1, 0.0313 – 313 hr-1, and 0.1 – 1000 µM, respectively.
